# What are the functional outcomes of total laryngeal transplantation? A systematic review of preclinical and clinical studies

**DOI:** 10.3389/fimmu.2025.1631525

**Published:** 2025-07-03

**Authors:** Douglas Henderson, Leonard Knoedler, Tobias Niederegger, Jakob Fenske, Olivier Mathieu, Gabriel Hundeshagen, Max Heiland, Curtis L. Cetrulo, D. Gregory Farwell, Jerome R. Lechien, Alexandre G. Lellouch

**Affiliations:** ^1^ Faculty of Medicine, Université Paris Cité, Paris, France; ^2^ Department of ENT and Head and Neck Surgery, Lariboisière Hospital, AP-HP, Paris, France; ^3^ Charité – Universitätsmedizin Berlin, Corporate Member of Freie Universität Berlin and Humboldt-Universität zu Berlin, Department of Oral and Maxillofacial Surgery, Berlin, Germany; ^4^ Division of Plastic and Reconstructive Surgery, Cedars-Sinai Hospital, Los Angeles, CA, United States; ^5^ Department of Hand, Plastic and Reconstructive Surgery, Burn Center, BG Trauma HospitalLudwigshafen, Ludwigshafen, Germany; ^6^ Department of Plastic and Hand Surgery, University of Heidelberg, Ludwigshafen, Germany; ^7^ Department of Otorhinolaryngology—Head and Neck Surgery, Hospital of the University of Pennsylvania and Perelman School of Medicine, Philadelphia, PA, United States; ^8^ Department of Surgery, University of Mons, Mons, Belgium; ^9^ Department of Otolaryngology-Head Neck Surgery, Foch Hospital, University of Paris Saclay, Paris, France; ^10^ Vascularized Composite Allotransplantation Laboratory, Center for Transplantation Sciences, Massachusetts General Hospital, Harvard Medical School, Boston, MA, United States; ^11^ Université Paris Cité, Inserm, The Paris Cardiovascular Research Center, Team Endotheliopathy and Hemostasis Disorders, Paris, France; ^12^ AP-HP, Hôpital Européen Georges Pompidou, Hematology Department, Paris, France

**Keywords:** laryngeal transplantation, allotransplantation, vascularized composite allografts, VCA, functional outcomes

## Abstract

**Purpose:**

This systematic review aims to evaluate the functional outcomes of total laryngeal transplantation by synthesizing findings from both preclinical and clinical studies. It focuses on assessing postoperative functional recovery, including swallowing, airway patency, phonation, and speech, while also considering the associated morbidities and immunosuppressive strategies.

**Methods:**

A systematic review was conducted for functional outcomes of total laryngeal transplantation through PubMed/MEDLINE, Embase, Scopus, and Web of Science databases according to Preferred Reporting Items for Systematic Reviews and Meta-Analyses guidelines. Case reports, case series, letters to the editor, reviews, and preclinical studies related to laryngeal transplantation were eligible for inclusion. Methodological quality and risk of bias were assessed via the CAMARADES checklist for preclinical studies and the JBI checklists for clinical studies.

**Results:**

Out of n=188 identified studies, n=16 (8.5%) met the inclusion criteria. There were n=13 (81%) clinical and n=3 (19%) preclinical studies. In preclinical models, canine and minipig studies showed partial recovery: electrical stimulation restored vocal fold mobility in n=8 (40%) of canine allografts; some minipigs recovered swallowing, vocalization, and short-term survival post-transplant without immunosuppression, though all canines remained tracheostomy-dependent. Among n=18 (100%) human recipients, speech or phonation was restored fully or partially in n=12 (67%), as well as full or partial oral intake. Here, n=3 patients (17%) died within two years post-VCA, while n=4 (36%) resumed full oral intake. Voice quality was considered as satisfactory or better than pre-VCA in n=6 (55%) patients, whereas airway patency was deemed good or excellent. Nonetheless, no patient regained full vocal fold mobility. However, n=1 (5.6%) patient was able to breathe without a tracheostomy, and n=1 (5.6%) could intermittently cap their tracheostomy tube. Immunosuppressive regimens included tacrolimus (n=18, 100%), mycophenolate mofetil (n=15, 83%), corticosteroids (n=15, 83%), and anti-thymocyte globulin (n=6, 33%), with adjunctive use of leflunomide and stem cells in select cases.

**Conclusion:**

Laryngeal transplantation shows promising results in restoring swallowing and phonation, but challenges remain for breathing without tracheostomy. The procedure remains an experimental surgery, still associated with significant morbidity and mortality, and requires lifelong immunosuppression. Future research, including long-term follow-up, larger-scale trials and interdisciplinary collaboration, is essential to further refine this procedure and evaluate its outcomes comprehensively.

## Graphical Abstract

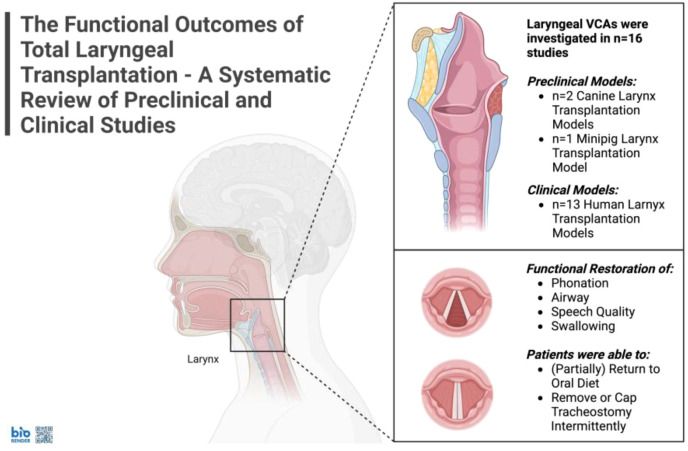

## 1 Introduction

The larynx is involved in major functions such as phonation, speech, swallowing, airway protection, and breathing (1). It is composed of various tissues, including muscles, cartilage, mucosa, nervous tissue, and connective tissue, making it a highly complex anatomical structure. Laryngeal transplantation is classified as a vascularized composite allograft (VCA), similar to hand (2), face (3, 4), or penile transplantation (5).

Total laryngectomy remains a high-volume surgical procedure, although laryngeal preservation strategies and nonsurgical treatments have grown increasingly important in the management of upper aerodigestive tract cancers (6). According to the World Health Organization, the global incidence of laryngeal cancer reached 189,191 new cases in 2022 (7). In the United States alone, around 5,000 total laryngectomies are performed annually (6). Besides oncological indications, this procedure may also be performed for severe trauma, stenosis, necrosis, post-radiotherapy complications, predisposition to chronic aspiration of food or recurrent laryngeal papillomatosis (8).

While total laryngectomy is generally considered safe (9), and significant advances have been made in rehabilitation strategies (10), losing one’s voice and living with a tracheostomy present substantial social and psychological challenges for patients and significantly impact their quality of life (11). Laryngeal transplantation has emerged as a potential life-enhancing treatment to restore function and form after total laryngectomy. Unlike life-saving organ transplants, such as those for the heart, lungs, or kidneys, this procedure is not essential for survival. Instead, its success is primarily assessed based on functional outcomes and associated morbidities. Thus, while it may not be sufficient on its own, precise microsurgical anastomosis of both vascular and neural structures is a necessary first step towards achieving satisfactory functional outcomes (12).

In 1928, American surgeon Franck Lahey laid the conceptual groundwork for laryngeal transplantation (13). However, the first human attempt—using a non-vascularized larynx—did not occur until 40 years later (14). It then took another 30 years to achieve the first successful human laryngeal transplantation (15). Throughout this timeline, animal studies have played a key role in refining surgical techniques, evaluating immunological tolerance, and assessing functional outcomes. These contributions have been instrumental in advancing the feasibility of laryngeal transplantation (16–20).

In this systematic review, the functional outcomes of laryngeal transplantation were examined, in both humans and preclinical models, highlighting key advancements and challenges. Furthermore, we discussed the post-operative rehabilitation strategies, immunosuppressive regimens, and future perspectives of this evolving field.

## 2 Methods

This systematic review was conducted following the Preferred Reporting Items for Systematic Reviews and Meta-Analyses (PRISMA) 2020 guidelines (21). Due to the heterogeneity of data, a qualitative synthesis approach was chosen, and a meta-analysis was deemed unsuitable. The study protocol was registered with PROSPERO (registration number CRD420250653746) (22).

### 2.1 Data source

An extensive literature search was performed using the Pubmed, Embase, Scopus, and Web of Science databases up to February 17^th^, 2025. The search strategy consisted of three key components combined via the Boolean operator “AND”: “larynx”, “transplantation”, and “functional outcome”. The full search strategy, including all synonyms and Medical Subject Headings (MeSH) terms.

### 2.2 Eligibility, inclusion, and exclusion criteria

For inclusion, case reports and case series had to be authored in English, French, Italian, or German. There were no restrictions on patient age or gender. Only patients who had undergone total laryngeal transplantation were eligible for inclusion. All clinical settings were included. Due to the paucity of literature, reviews, comments and letters to the editor were considered eligible for inclusion. Conference abstracts, book chapters, non-VCA clinical studies, and anatomical or feasibility studies were excluded. Preclinical studies of any type were eligible for inclusion.

### 2.3 Study selection process

**Figure 1:**
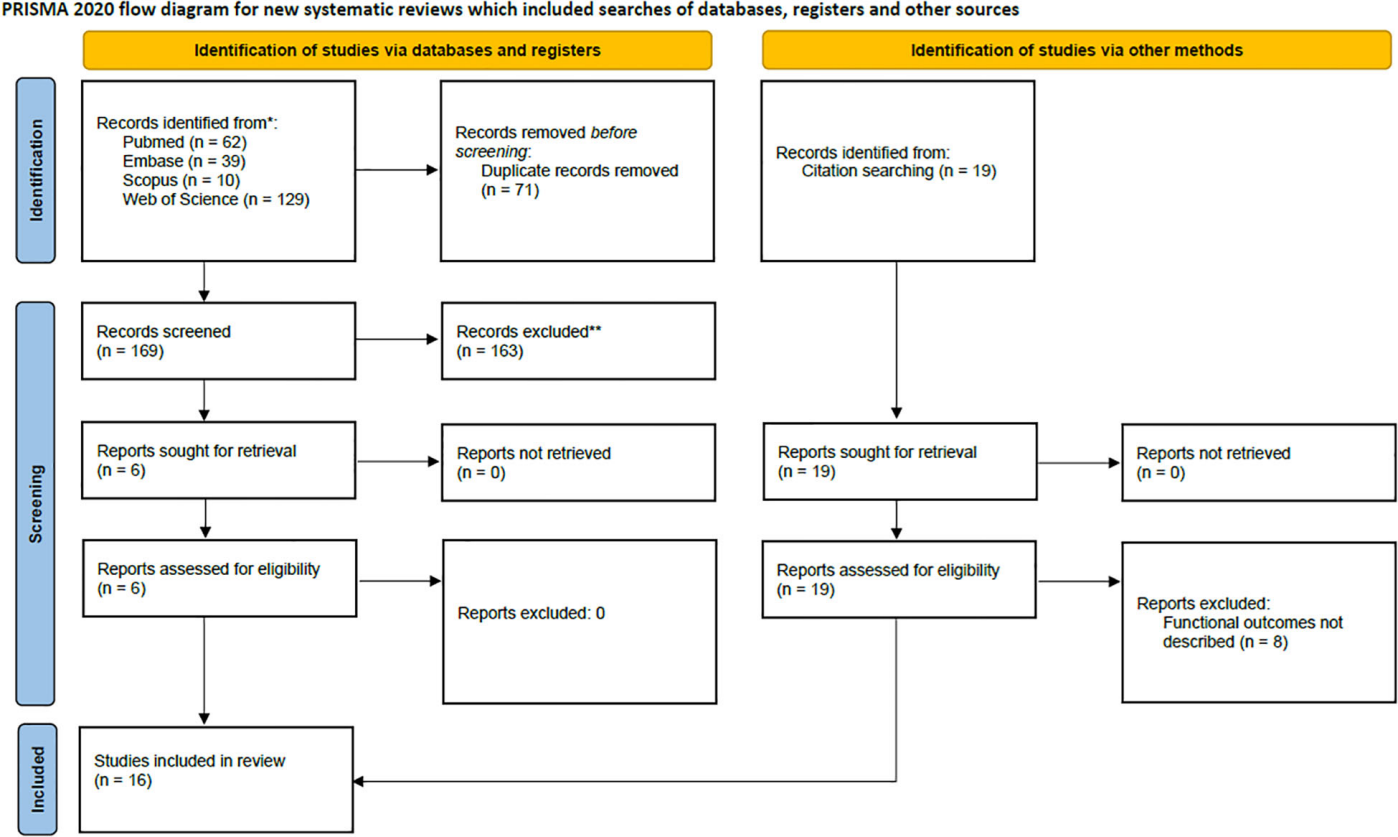
PRISMA flow chart.

Title and abstract screenings were independently carried out by two reviewers (DH and LK), followed by a thorough assessment of all eligible full-text articles. Studies were evaluated for relevance based on the predefined inclusion and exclusion criteria. Any disagreements during the selection process were resolved through consultation with a third reviewer (JF) to ensure consensus. Further details are provided in **Figure 1**.

### 2.4 Data extraction

The following study characteristics were extracted using a standardized Excel spreadsheet: first author, year of publication, species, number and type of grafts, mobility of the vocal cords (significant or volitional), swallowing, breathing (definitive decannulation or closing of the tracheostomy), phonation, speech (in humans), number of rejections, rejection assessment methods, induction and maintenance of immunosuppressive treatments, follow-up methods and timing. Data was extracted from text, figures, tables, and graphs.

### 2.5 Quality assessment

The quality and risk of bias in preclinical animal studies including a control group were assessed using the Collaborative Approach to Meta-Analysis and Review of Animal Experimental Studies (CAMARADES) checklist (23). The CAMARADES checklist typically includes 10 items that assess the methodological quality of preclinical studies using animal models. These items evaluate key aspects such as randomization, blinding, sample size calculation, and bias control. Each item is assigned a score (often 0 or 1), and the total score reflects the overall quality of the study. Higher scores indicate better methodological rigor and higher study quality. Lower scores suggest a higher risk of bias and lower reliability of the results. The Joanna Briggs Institute Critical Appraisal Checklists assess the quality of case reports and case series based on clarity, methodology, and result reporting (24). They include 8 items for case reports and 10 items for case series. A high score indicates better quality and lower risk of bias, while a low score suggests lower reliability (25).

## 3 Results

### 3.1 General study characteristics

The literature search resulted in a total of n=188 studies, of which n=16 (98.5%) were included. Case reporting quality, assessed using the JBI checklists in n=8 (50%), was deemed very satisfactory. Among the analyzed preclinical studies, n=3 (19%), only n=1 (6.3%) met the required criteria for the CAMARADES assessment and received a low score (3/10). Further details on quality assessment are provided in **Table 1**.

**Table 1:** Quality and risk of bias assessment according to Joanna Briggs Institute (JBI) critical appraisal checklists.

First author	Publication year	Q1	Q2	Q3	Q4	Q5	Q6	Q7	Q8	Q9	Q10	Type of article
Marshall S. Strome	2001											Case report
Robert R. Lorenz	2004											Case report
Samir S. Khariwala	2007											Narrative review
P. Daniel Knott	2011											Letter to the Editor
D. Gregory Farwell	2013											Case report
Robert R. Lorenz	2014											Commentary*
Quang Luu	2014											Narrative review
Maciej Grajek	2017											Case report
Giri Krishnan	2017											Systematic review
Estephania Candelo	2024											Case series
Mailudan Ainiwaer	2024											Case report
Philippe Céruse	2024											Letter to the Editor
David G. Lott	2024											Case report

green: yes, orange: unclear, red: no, grey: not applicable.

*we deemed it reasonable to apply the same evaluation criteria as those used for case reports.

### 3.2 Functional outcomes in preclinical studies

Overall, n=3 (19%) studies used animal models, including n=2 (13%) canine models and n=1 (6%) minipig model. Starting with canine autografts, Broniatowski et al. investigated the reinnervation of the intrinsic muscles of the larynx. The authors found that n=8 (40%) of the study animals exhibited mobility of the vocal folds in response to external electrical stimulation (18).

Furthermore, Anthony et al. performed laryngeal autografts, as well as orthotopic and heterotopic allografts. In the autograft group, dogs were re-implanted with their own larynx, while in the allografts groups, dogs were implanted with a larynx from another animal, either in place of their own larynx (orthotopic) or adjacent to their own larynx (heterotopic), which remained intact. On postoperative day 60, adduction of the vocal folds following tracheal and cord mechanical stimulation was observed only in n=1 (33%) animal from the autograft group. This animal showed glottic competence, which allowed a satisfactory swallowing function. However, all dogs remained tracheostomy-dependent, while the authors did not include any data on phonation assessment. One subject in the orthotopic allograft group survived for 33 days, whereas both subjects in the heterotopic allograft group survived beyond 60 days. Regarding immunosuppressive regimens, dogs received treatments such as cyclosporin A, mycophenolate mofetil, and methylprednisolone (17).

Conversely, in the minipig model, Birchall et al. restored the ability to swallow in n=3 (18%) out of n=17 allografts, as assessed by video-fluoroscopy. A total of n=7 (41%) animals demonstrated laryngeal airflow when the T-tube tracheotomy was occluded, and n=4 (24%) animals were able to produce audible grunts. However, minipigs were fully MHC II-matched and received no immunosuppressive treatment. Overall, n=7 (41%) minipigs survived and were alive at 7 days post-laryngeal VCA (19). Full details of functional outcomes in animal models are shown in **Table 2**.

**Table 2:** Data synthesis from preclinical studies.

First author	Year of publication	Animal model	Number and type of grafts	Mobility of the vocal cords (significant or volitional)	Swallowing	Breathing (definitive decannulation or closing of tracheostomy)	Phonation	Speech	Number of rejections	Rejection assessment methods	Induction and maintenance of immunosuppressive treatments	Follow up methods and timing
Michael Broniatowski (18)	1989	Dog	20 autografts	No, only via external electrical stimulation (observed in all n=8 survivors out of n=20)	Not explicitly mentioned	No	Not explicitly mentioned	NA	NA	NA	NA	Endoscopic examination, oscilloscope and chart recorder
J. E Anthony (17)	1995	Dog	3 autografts	No	Yes, in n=1 survivor out of n=3	No	NA	NA	NA	NA	NA	Daily inspections
			6 orthotopic allografts	No	NA	No	NA	NA	Orthotopic group: n=1 survivor out of n=6 showed clear signs of graft rejection (CsA alone)	Clinical evaluation	Orthotopic allografts (n=3 out of n=6): CsA	weekly biopsies
			2 heterotopic allografts	No	NA	NA	NA	NA	No rejection episode	biopsy, mixed lymphocyte cultures	Orthotopic allografts (n=3 out of n=6) and heterotopic allografts: CsA, RS-61443, methylprednisolone	weekly CsA level monitoring
M. A. Birchall (erratum) (26) M. A. Birchall (19)	2011	Minipig	17 allografts	Not explicitly stated	Yes, n=3 out of n=4 videofluoroscopy-cooperative pigs, among the n=7 survivors at 1 week	No	Yes, n=5 out of the n=7 survivors at 1 week	NA	Not explicitly stated, n=8 graft failures	Clinical evaluation, laser doppler fluxmetry, endoscopy, biopsy, myology assessment	None, “fully matched pigs at the MHC2 loci”	Endoscopy at 48 hours and 7 days, (videofluoroscopy), barium swallow, airflow measurement

NA, not applicable; CsA, Cyclosporine A.

### 3.3 Functional outcomes in human VCAs

Laryngeal VCAs in human patients were reported in n=13 (81%) studies. The first case was performed on a male patient in 1998 in Cleveland by Strome et al. (15, 27–30). The male patient had suffered for two decades from post-traumatic laryngeal dysfunction, including aphonia, after his larynx and pharynx were crushed in a motorcycle accident. Three years post-VCA surgery, the authors described the patient’s voice and speech quality as “*within the normal range*” and reported a patient-perceived improvement in quality of life. Phonation parameters were assessed in terms of maximum phonation time, intensity, and airflow. The patient’s swallowing ability was described as “*outstanding*”. Nonetheless, the patient remained tracheostomy-dependent, and his vocal folds never regained satisfactory mobility. At last, chronic rejection led to the removal of the allograft 14 years post VCA surgery. The patient was maintained on cyclosporine, mycophenolate mofetil, and prednisone; perioperative regimens also included muromonab-CD3 and azathioprine.

A female laryngeal VCA case was performed by Farwell et al. in 2012 in California (31, 32). The patient had suffered from complete laryngotracheal stenosis for 11 years and was on lifelong immunosuppression due to a prior kidney-pancreas transplant. Post VCA, she was able to resume a standard oral diet and was very satisfied with her voice. She underwent an extensive vocal assessment (i.e. fundamental frequency, maximum phonation time, noise-to-harmonic ratio, and vocal frequency and intensity range). However, her voice did not reach fully normal acoustics parameters. No vocal fold mobility or decannulation were achieved. Her immunosuppression included induction with rabbit anti-thymocyte globulin, methylprednisolone, and mycophenolate mofetil, followed by maintenance with tacrolimus and leflunomide.

Similarly, Grajek et al. performed laryngeal VCA on an immunosuppressed male kidney transplant recipient in 2015 in Poland (12). The patient presented with a squamous cell carcinoma of the larynx, resulting in total laryngectomy. Two years post VCA, the patient’s vocal cords were described as “*functioning*”. His swallowing and speech were restored. Notably, he was able to breathe without tracheostomy. He received induction with anti-thymocyte globulin, tacrolimus, and methylprednisolone, and remained on tacrolimus, methylprednisolone and mycophenolate mofetil.

Candelo et al. presented the largest case series with 11 laryngeal VCAs, including the three patients mentioned above, an additional three patients from Poland, and five from Colombia (33). By the two-year follow-up, n=3 (27%) patients were deceased. Notably, n=1 (9.1%) patient died due to pulmonary embolism on postoperative day 1 and n=2 (18%) due to sepsis after six and 18 months, respectively. In the surviving patients, n=4 (36%) had a strong enough swallowing function to be on a fully oral diet. Voice quality was absent in n=1 (9.1%), poor in n=1 (9.1%), satisfactory in n=3 (27%), good in n=1 (9.1%), or excellent in n=2 (18%). At the same time, airway patency was absent in n=2 (18%) patients and good or excellent for the remaining n=6 (55%). Immunosuppression varied but frequently included combinations of prednisone, methylprednisolone, tacrolimus, mycophenolate mofetil, and anti-thymocyte globulin, with some protocols incorporating mesenchymal stem cells.

Ainiwaer et al. reported the first Chinese laryngeal transplantation in April 2023, with a six-month follow-up. The patient had undergone a partial laryngectomy nine years earlier due to recurrent laryngeal squamous cell carcinoma. After laryngeal VCA, swallowing was partially restored, and the patient was on a semi-liquid oral diet. He had a tracheostomy and was able to speak (34). The patient was treated with tacrolimus, methylprednisolone, and mycophenolate mofetil.

The first French laryngeal transplant was performed in September 2023. The female patient had been unable to speak for two decades. However, post VCA, the patient regained the ability to speak, while still requiring a tracheotomy cannula and a gastrostomy tube (35). Interestingly, Céruse et al. reported that, beyond neural micro-sutures, they had also performed two neurotizations to possibly improve functional outcomes. Neither the immunosuppressive regimen nor the details of the surgical procedure were reported.

The latest case was performed in a patient with active cancer (i.e., low-grade laryngeal chondrosarcoma) in the United States in February 2024. The patient was a kidney transplant recipient on lifelong immunosuppression. Lott et al. reported that a few months after surgery, the patient regained the ability to swallow liquids and solids without limitation but still needed his gastric feeding tube to receive a sufficiently high volume. The patient’s voice quality was estimated at 60% of normal, and he was able to cap his tracheostomy tube intermittently (36). He remained on lifelong immunosuppression, although specific agents were not detailed.

Further insights into the functional outcomes of human VCAs, and additional data, including immunosuppressive regimens, are presented in **Table 3**.

**Table 3:** Data synthesis from clinical studies.

First author	Publication date	Number and type of grafts	Mobility of the vocal cords (significant or volitional)	Swallowing	Breathing (definitive decannulation or closing of the tracheostomy)	Phonation	Speech	Number of rejections	Rejection assessment methods	Induction and maintenance of immunosuppressive treatments	Follow up methods and timing
Marshall S. Strome (27)	2001	1 allograft	No	Yes	No	Yes	Yes	1 episode	Clinical evaluation, observation of voice changes, biopsies, endoscopic examination	Before surgery: cyclosporine, azathioprine, and methylprednisolone. Induction phase: muromonab-CD3, cyclosporine, methylprednisolone, mycophenolate mofetil Maintenance phase: Cyclosporine, mycophenolate mofetil, and prednisone At the time of publication: prednisone, mycophenolate mofetil, tacrolimus	Endoscopic and histological examination, monitoring of drug plasma concentrations, clinical assessment, voice testing, electromyography, barium swallow
Robert R. Lorenz (28)	2004	1 allograft	No	Not explicitly mentioned	No	Yes	Yes	Not explicitly mentioned	Not explicitly mentioned	Not explicitly mentioned	Clinical assessment, endoscopic examination, voice testing, electromyography
Samir S. Khariwala (30)	2007	1 allograft	No	Yes	Not explicitly mentioned	Yes	Yes	2 episodes (15 months, and 6 years)	Clinical evaluation, endoscopic examination, biopsy	Before surgery: cyclosporine, azathioprine, and methylprednisolone Induction phase: muromonab-CD3, cyclosporine, methylprednisolone, mycophenolate mofetil Maintenance phase: prednisone, mycophenolate mofetil, tacrolimus	Not explicitly stated, iodine-123 uptake scans, blood tests (calcium and phosphate), electromyography, barium swallow, voice testing
P. Daniel Knott (29)	2011	1 allograft	No	Yes	No	Yes	Yes	4 episodes, including “chronic rejection”	Clinical evaluation, endoscopic examination, endoscopic videostroboscopy	Induction phase: cyclosporine Maintenance Phase: Tacrolimus, Prednisone, Mycophenolate mofetil	Serum levels of tacrolimus, bone densitometry, barium swallow evaluation, voice testing
D. Gregory Farwell (31)	2013	1 allograft	No	Yes	No	Yes	Yes	No rejection episode	Clinical evaluation, biopsy, solid-phase assay (donor-specific anti-HLA antibodies)	Immunosuppressive regimen related to prior transplantation: tacrolimus and leflunomide Induction phase: rabbit anti-thymocyte globulin, methylprednisolone, mycophenolate mofetil, (tacrolimus) Maintenance phase: tacrolimus, leflunomide	Daily endoscopic inspections from day 1-8, 10, 14, and 20. Biopsies were taken 6 hours post-transplant and on PODs 1, 14, 30, and 137. Donor specific anti-HLA antibodies (solid-phase assay) at 6 months, voice testing, swallowing assessment
Robert R. Lorenz (15)	2014	1 allograft	No	Yes	No	Yes	Yes	“Starting around the twelfth year after transplantation, a combination of an infectious and chronic rejection pattern emerged.”	clinical evaluation, biopsy	Maintenance phase: prednisone, tacrolimus, mycophenolate mofetil	I-123 uptake scan, voice testing, swallowing assessment, biopsy
Quang Luu (32)	2014	2 allografts	No	Yes	No	Yes	Yes	None reported	Clinical evaluation, Laryngeal biopsies	Immunosuppressive regimen related to prior transplantation: tacrolimus and leflunomide	Biopsies taken on postoperative days 1, 14, 30, and 137, electromyography, not explicitly stated
Maciej Grajek (12)	2017	1 allograft	No, yet described as “functioning”	Yes	Yes	Yes	Yes	No rejection episode	Clinical evaluation, endoscopic examination, blood markers (PTH, TSH, FT3, FT4, and calcium blood levels monitoring), large skin paddle clinical evaluation	Induction phase: anti-thymocyte globulin (thymoglobulin), tacrolimus, mycophenolate mofetil, methylprednisolone Maintenance phase: tacrolimus, mycophenolate mofetil, methylprednisolone	Follow-up assessment was performed monthly and consisted of endoscopical vocal chords movement evaluation, PTH, TSH, FT3, FT4, and calcium blood levels monitoring, and data acquisition directly from the patient (breathing/swallowing). After 2 years, assessments were performed every 4 months. Thyroid scintigraphy
Giri Krishnan (37)	2017	2 allografts	No No	Yes Yes	No No	Yes Yes	Yes Yes	Several episodes No rejection episode	Clinical evaluation, endoscopic examination, biopsy	Induction phase: muromonab-CD3, cyclosporine, methylprednisolone, and mycophenolate mofetil Maintenance phase: prednisone, mycophenolate mofetil, and tacrolimus Immunosuppressive regimen related to prior transplantation: tacrolimus and leflunomide Induction phase: rabbit antithymocyte globulin, methylprednisolone, mycophenolate mofetil, (tacrolimus) Maintenance phase: tacrolimus and leflunomide	Not explicitly mentioned
Estephania Candelo (33)	2024	11 allografts	No	Yes, at two years: n=1 “Highly restricted oral intake”, n=3 “Partial oral intake”, n=4 “Fully oral intake” (among n=8 survivors)	Yes and No, airway patency at two years: N=2 “absent”, n=2 “good”, n=4 “excellent” “Only two patients at 2 years (were) retaining a form of tracheal stoma” and “those decannulated (n=4 out of n=8 recipients reaching 2 years follow up)” (among n=8 survivors)	Yes and No, at two years: n=7 out of n=11 (among n=8 survivors)	Yes and No, at two years: N=1 “absent”, n=1 “poor”, n=3 “satisfactory”, n=1 “good”, n=2 “excellent” (among n=8 survivors)	Early rejection: n=3 Chronic rejection: n=4 Partial graft excision: n=2 Total graft excision: n=1 Structural changes in the allograft: n=4 (among n=8 survivors)	Clinical evaluation, endoscopic examination, biopsy, stroboscopy, laryngeal electromyography	Various regimens using prednisone, methylprednisolone, anti-thymocyte globulin, cyclosporine, azathioprine, mycophenolate mofetil, daclizumab, tacrolimus, sirolimus, and mesenchymal stem cells	Various protocols For the “first case”: “monthly for 6 months, followed by every 3 months with endoscopy and yearly biopsy”
Mailudan Ainiwaer (34)	2024	1 allograft	No	Yes	Not explicitly stated (probably not)	Yes	Yes	No rejection episode	Clinical evaluation, endoscopic examination, blood markers including cytokines, thyroid biopsy	methylprednisolone, tacrolimus, and mycophenolate mofetil	Not explicitly stated, swallowing assessment, voice testing, clinical evaluation, endoscopic examination
Philippe Céruse (35)	2024	1 allograft	Not explicitly stated (probably not)	Not explicitly stated	No	Yes	Yes	No rejection episode	Not explicitly stated	Not explicitly stated	Not explicitly stated
David G. Lott (36)	2024	1 allograft	Not explicitly stated (probably not)	Yes	No	Yes	Yes	No rejection episode	Not explicitly stated	Not explicitly stated, the patient was already on immunosuppression from a previous kidney transplant	Not explicitly stated

NA, not applicable; MHC, Major Histocompatibility Complex; PTH, Parathyroid Hormone; TSH, Thyroid-Stimulating Hormone; FT3, Free Triiodothyronine; FT4, Free Thyroxine; I-123, Iodine-123; CD3, Cluster of Differentiation 3; POD, Post-Operative Day.

## 4 Discussion

Laryngeal transplantation represents a type of VCA to restore laryngeal function. Currently, functional outcomes are encouraging in terms of swallowing, phonation, and speech, but vocal fold mobility and tracheostomy dependence persist as main barriers in the field. Furthermore, laryngeal transplantation is a procedure associated with significant morbidity and mortality.

Our synthesis of all published cases suggests that voluntary mobility of the vocal folds was never fully restored. Swallowing function, even if minor, was reported in most patients, while some no longer required a tracheostomy. Additionally, two-thirds of the patients regained phonatory and speech functions, fully or partially.

It is naturally challenging to compare functional outcomes across different types of VCA, as they restore inherently distinct functions. Nevertheless, it would appear that functional recovery in other VCA types has generally exceeded that seen in laryngeal VCA. In upper extremity VCA, studies report consistent return of protective sensation, voluntary movement, and, in many cases, fine motor skills within 12–24 months – (38–40). These outcomes are considered reproducible across centers and often surpass prosthetic alternatives (41, 42). Furthermore, early rehabilitation and interdisciplinary support were also shown to critically enhance functional outcomes and long-term success (43). Similarly, facial VCA has shown recovery of expression, speech, and psychosocial integration, with early sensory return and functional gains that exceed traditional reconstruction (44–46). Other VCA types, such as uterus, trachea, and abdominal wall have demonstrated key functional outcomes including live births, airway patency, and structural support (41, 47, 48).

Laryngeal VCA may benefit from validated functional outcome measures, adapted rehabilitation protocols, and multicenter collaboration to optimize results. Incorporating patient-reported outcomes and identifying biomarkers for graft function and rejection could further support long-term success.

Identifying causes of rejection and mortality is essential to improving the safety of any experimental procedure. In their 2017 review, Krishnan et al. outlined complications in the first two laryngeal transplant recipients: the first developed multiple infections (oropharyngeal candidiasis, tracheobronchitis, Pneumocystis carinii pneumonia), acute rejection at 15 months and 5 years, and signs of chronic rejection at 9 years, leading to graft explantation at 14 years. The second patient experienced mild infectious complications (pulmonary and catheter-related infections, mucosal candidiasis), and tacrolimus toxicity, with no rejection at 6 months (37). In a case series by Candelo et al., three of eleven patients died within 20 years—one from pulmonary embolism and two from sepsis—despite having functioning grafts. Complications were frequent but typical of major surgery, including early pneumothorax, perioperative hemorrhage, and late airway stenosis (33).

Reported causes of rejection and mortality in early laryngeal transplantation include opportunistic infections, immunosuppressive toxicity, and progressive chronic rejection, whereas deaths were primarily attributed to indirect postoperative complications such as pulmonary embolism and sepsis, rather than graft failure itself.

An essential factor in preventing graft rejection and patient mortality is the optimization of immunosuppressive therapies. Our study found corticosteroids (prednisone, methylprednisolone), antibodies (anti-thymocyte globulin, daclizumab), calcineurin inhibitors (cyclosporine, tacrolimus), antimetabolites (azathioprine, mycophenolate mofetil), and mTOR inhibitors (sirolimus), to be the most common immunosuppressants in laryngeal VCA. Mesenchymal stem cells were used in select cases. In the literature, Baudouin et al. analyzed the first three laryngeal VCA cases, noting a shift from high-dose cyclosporine to lower-dose rabbit anti-lymphocyte globulin and tacrolimus. Mycophenolate mofetil became standard, and prednisone remained effective across transplant types (49). In comparison in facial VCA, Huelsboemer et al. reported that facial VCA induction regimens typically consisted of corticosteroids, thymoglobulin, tacrolimus, and mycophenolate mofetil, with maintenance relying on tacrolimus, mycophenolate mofetil, corticosteroids, and extracorporeal photopheresis. In hand transplantation similar approaches were reported. Here, immunosuppressive regimens often included basiliximab and mTOR inhibitors such as sirolimus or everolimus (50). These parallels illustrate a shared immunosuppressive backbone across VCA types, emphasizing a core reliance on calcineurin inhibitors and antimetabolites, while also showcasing the progressive integration of adjunctive therapies like mTOR inhibitors and biologics. Notably, the inclusion of extracorporeal photopheresis in facial VCA highlights efforts to modulate the immune system while minimizing systemic toxicity. Moreover, hand VCA studies have explored steroid-sparing regimens and demonstrated successful tolerance protocols using costimulation blockade, pointing to future opportunities in laryngeal VCA. Looking forward, Baudouin et al. emphasized the importance of standardized immune surveillance, including pre-transplant crossmatching, anti-HLA antibody monitoring, and Luminex-based single-antigen assays (49). In experimental models, Lott et al. proposed pulsed everolimus in combination with anti–αβ T-cell receptor antibodies to reduce long-term toxicity, presenting a novel, less aggressive immunosuppressive strategy tailored to oncologic laryngeal transplants (51).

These findings suggest clinicians should re-evaluate immunosuppressive strategies in laryngeal VCA by drawing on advances from other VCA types. The successful application of maintenance strategies from facial and hand VCAs, including extracorporeal photopheresis or mTOR inhibitors, shows promise for adaptation. Furthermore, standardizing immune monitoring protocols—like HLA antibody tracking and single-antigen assays—could enhance early detection and management of rejection. Ultimately, translating these insights into clinical practice may help reduce morbidity, prolong graft survival, and enable broader adoption of laryngeal VCA.

Living without a larynx is not inherently life-threatening. Consequently, the risks associated with lifelong immunosuppression, such as increased susceptibility to malignancy (52), complex infection management (53), and the possibility of allograft rejection must be weighed carefully on a case-by-case basis. These concerns have been extensively addressed in the literature over the past two decades (26, 37, 54–56). Therefore, rigorous and validated assessment of functional outcomes in laryngeal transplantation is ethically imperative to ensure that patients can make fully informed decisions. Moreover, the issue of potential retransplantation, as has been encountered in facial transplantation, must be considered (44, 57). Laryngeal transplantation shares many systemic challenges with other forms of organ transplantation, including high treatment costs, variable insurance reimbursement, and limited graft availability. Importantly, demographic data, particularly regarding ethnicity and race, are not consistently reported. Institutions performing laryngeal transplantation should implement measures to ensure equitable access, such that recipients reflect the demographic diversity of the broader population. For example, in the series reported by Candelo et al., only 27% of recipients were women, with 9% identifying as Black, 45% as Hispanic, and 45% as White (33), an imbalance similarly noted in facial transplantation research (58).

Overall, clinical experience with laryngeal transplantation remains scarce. Therefore, multiple authors provided valuable insights on alternative approaches for larynx reconstruction. Here, Andrews et al. investigated hemilaryngeal transplantation (HLT) in a canine model as a solution for partial laryngeal defects, demonstrating that HLT was well-tolerated with successful revascularization and reinnervation of the thyroarytenoid muscle, leading to symmetric phonation and viable tissue (59). Supracricoid laryngectomy (SCL) may avoid the need for a permanent tracheostomy but often results in speech and swallowing deficits, comparable to those of a total laryngectomy. Zacharek et al. reported similar functional limitations in both laryngeal VCA and SCL (60). Looking into radial forearm flap reconstructions (RFFR) after total laryngectomy, functional outcomes were similar to laryngeal VCA. However, RFFR patients showed lower Voice-Related Quality of Life scores (i.e. a scale to assess the functional impact of voice disorders on daily activities) (61). Additional emerging modalities such as transoral robotic surgery (TORS) and tracheoesophageal voice prostheses have further expanded reconstructive possibilities. TORS offers a minimally invasive method for tumor resection with improved preservation of swallowing and voice (62), while voice prostheses remain a widely used solution for communication after laryngectomy (63, 64). Together, these findings supported the notion that laryngeal VCA might offer functional restoration that is at least comparable, if not superior, to conventional approaches, while maintaining a similar risk profile.

For clinicians, this suggests that laryngeal transplantation should be considered a viable reconstructive option in carefully selected patients, particularly those with traumatic or oncologic defects where conventional techniques would result in significant functional limitations. Despite challenges with vocal fold mobility, many patients regain swallowing, phonation, and speech, suggesting meaningful benefit. Outcomes may rival or exceed those of SCL or RFFR, especially in terms of quality of life. Given the high morbidity and resource intensity, strict patient selection and multidisciplinary planning remain critical. Patients should be counseled on the potential for improved voice and airway function, but also on the lifelong immunosuppression and current uncertainties in rejection monitoring, emphasizing the importance of long-term follow-up in specialized centers.

## 5 Limitations

A key methodological limitation is the uncertain number of laryngeal transplants worldwide, for instance Krishnan et al.’s 2017 review found only two documented cases. They estimated at least 14 cases since 2002, based on Spanish-language abstracts, notably by Duque, Tinitinago, and Terris (37). It is highly probable that five of these cases correspond to those reported from Colombia in the 2024 case series by Candelo et al. (33). However, the reasons why the remaining nine cases were not included remain unclear. The scarcity of documented cases limits the ability to conduct large-scale comparative studies and calls for the establishment of an international registry to improve data collection.

Second, the articles included in this systematic review are not limited to original research papers, as is commonly the case in this type of research. The heterogeneity of sources (reviews, case series, commentaries, etc.) also poses a methodological limitation. This approach was necessitated by the limited availability of data and was also the strategy adopted in the systematic review conducted by Krishnan et al. (37).

Third, in the case reports and case series included in this review, data from standardized and reproducible evaluation tools for assessing vocal fold mobility, phonation, speech, swallowing, and airway patency were not consistently available, making direct comparisons between cases difficult. Combining validated questionnaires and objective tools would better capture both patient and clinician perspectives. This calls for the establishment of an international database to improve data collection, facilitate more consistent functional outcome assessments, and encourage long-term follow-ups. It would allow for large-scale comparative studies and meta-analyses.

Lastly, due to the experimental nature and high technical complexity of this procedure, early failures may have gone unreported in the literature. This inevitably introduces a potential reporting bias that is difficult to quantify when conducting descriptive statistical analyses based on case series. Establishing an international prospective registry is essential to address this limitation and improve data transparency.

## 6 Conclusion

Laryngeal transplantation is a type of VCA that can help restore the function and the form of the larynx. Larynx allotransplants show promising effects in improving the patient’s swallowing and phonation. However, significant limitations persist. To date, no recipient has regained full vocal fold mobility, and sustained decannulation remains rare, highlighting persistent deficits in neural reinnervation and airway autonomy. These functional constraints, combined with the burden of lifelong immunosuppression and associated morbidity, confine the procedure to an experimental domain. Regarding future research, three domains should be prioritized: first, the development of standardized, reproducible outcome measures that integrate objective functional data with patient-reported experiences, second, the optimization of surgical techniques and rehabilitation protocols through multicenter collaboration, and third, the exploration of novel immunomodulatory strategies aimed at reducing long-term systemic toxicity while preserving graft viability. A retrospective case-control study comparing functional outcomes between transplantation and non-transplantation controls after total laryngectomy could provide valuable insights, reinforcing the potential advantages of laryngeal transplantation and underscoring the necessity for further research in this field. Furthermore, recent advances in predictive modeling and early rejection diagnosis developed in the context of solid organ transplantation could be applied to laryngeal VCA, including those based on artificial intelligence. Such technologies could help improve the long-term management of patients.

At present, laryngeal transplantation is still considered an experimental procedure for select indications in specific patients. Its ethical justification depends on its capacity to deliver strong and sustainable improvements in quality of life. To better evaluate the functional outcomes and morbidity of laryngeal transplantation, patients should be followed over longer periods and assessed using standardized tools. Overall, this work calls for future cross-disciplinary research and international registers to determine the need for laryngeal transplants and better document outcomes over time.
